# Resting State BOLD Variability in Alzheimer’s Disease: A Marker of Cognitive Decline or Cerebrovascular Status?

**DOI:** 10.3389/fnagi.2018.00039

**Published:** 2018-02-21

**Authors:** Vanessa Scarapicchia, Erin L. Mazerolle, John D. Fisk, Lesley J. Ritchie, Jodie R. Gawryluk

**Affiliations:** ^1^Department of Psychology, University of Victoria, Victoria, BC, Canada; ^2^Department of Radiology and The Hotchkiss Brain Institute, University of Calgary, Calgary, AB, Canada; ^3^Department of Psychology and Neuroscience, Dalhousie University, Halifax, NS, Canada; ^4^Department of Psychology, Nova Scotia Health Authority, Queen Elizabeth II Health Sciences Centre, Halifax, NS, Canada; ^5^Department of Psychiatry, Dalhousie University, Halifax, NS, Canada; ^6^Department of Psychology and Neuroscience, Dalhousie University, Halifax, NS, Canada; ^7^Department of Medicine, Dalhousie University, Halifax, NS, Canada; ^8^Department of Clinical Health Psychology, University of Manitoba, Winnipeg, MB, Canada

**Keywords:** signal variability, Alzheimer’s disease, biomarker, BOLD fMRI, aging

## Abstract

**Background:** Alzheimer’s disease (AD) is a neurodegenerative disorder that may benefit from early diagnosis and intervention. Therefore, there is a need to identify early biomarkers of AD using non-invasive techniques such as functional magnetic resonance imaging (fMRI). Recently, novel approaches to the analysis of resting-state fMRI data have been developed that focus on the moment-to-moment variability in the blood oxygen level dependent (BOLD) signal. The objective of the current study was to investigate BOLD variability as a novel early biomarker of AD and its associated psychophysiological correlates.

**Method:** Data were obtained from the Alzheimer’s Disease Neuroimaging Initiative (ADNI) 2 database from 19 participants with AD and 19 similarly aged controls. For each participant, a map of BOLD signal variability (SD_BOLD_) was computed as the standard deviation of the BOLD timeseries at each voxel. Group comparisons were performed to examine global differences in resting state SD_BOLD_ in AD versus healthy controls. Correlations were then examined between participant SD_BOLD_ maps and (1) ADNI-derived composite scores of memory and executive function and (2) neuroimaging markers of cerebrovascular status.

**Results:** Between-group comparisons revealed significant (*p* < 0.05) increases in SD_BOLD_ in patients with AD relative to healthy controls in right-lateralized frontal regions. Lower memory scores and higher WMH burden were associated with greater SD_BOLD_ in the healthy control group (*p* < 0.1), but not individuals with AD.

**Conclusion:** The current study provides proof of concept of a novel resting state fMRI analysis technique that is non-invasive, easily accessible, and clinically compatible. To further explore the potential of SDBOLD as a biomarker of AD, additional studies in larger, longitudinal samples are needed to better understand the changes in SDBOLD that characterize earlier stages of disease progression and their underlying psychophysiological correlates.

## Introduction

Alzheimer’s disease is a progressive, neurocognitive disorder characterized by impairments in memory, as well as other cognitive domains, including language, visuospatial skills, and executive functions (Alzheimer’s Association, 2016). Although a number of factors have been associated with the development of AD, epidemiological evidence suggests that the strongest risk factor for AD is age. Nearly one out of every nine individuals beyond the age of 65 is expected to develop the disease; by age 85, this figure rises markedly to an estimate of nearly one out of three (Alzheimer’s Association, 2016). In light of globally increasing life expectancies and a rapidly aging population, AD has become an urgent public health concern (Winblad et al., 2016).

At present, there are no curative treatments for AD (Scheltens et al., 2016). Available treatment options are limited and focus primarily on delaying the progression of symptoms (Wilkinson, 2012; Alzheimer’s Association, 2016). There are a number of lifestyle factors, both reversible and irreversible, that may increase or reduce an individual’s risk for developing dementia. It has recently been proposed that up to a third of all cases of dementia may theoretically be prevented through population-level interventions targeted at modifiable risk factors (Livingston et al., 2017). While there is limited evidence to support the use of mass cognitive screens due to their unclear benefits, improving the identification of high-risk groups will be imperative in order to deliver targeted treatment. This points to an ever-increasing need for the identification of reliable, early biomarkers for AD.

The ideal technique for biomarker identification would be non-invasive, easily repeatable, and widely available, as is MRI. Although most MRI based biomarker research on AD to date has focused on structural changes in gray matter (GM; Cash et al., 2014), MRI can also provide relevant measures of brain function. BOLD functional MRI (fMRI) is a MRI based technique that allows for non-invasive examination of brain function by measuring fluctuations in signal intensity over time that are a consequence of oxygenated blood supplying active neurons. Recently, resting-state fMRI (rsfMRI) has emerged as a promising clinical imaging method, as it eliminates the cognitive burden of task performance that is characteristic of task-based fMRI and thus reduces the level of compliance required of the patient (Fox and Greicius, 2010; Mueller et al., 2012).

Traditionally, the majority of fMRI investigations have based their findings on patterns of *mean* brain activity. This is based on the longstanding premise that the mean value across an fMRI time-series represents the average, and therefore most representative, “signal” among a distribution of unwanted “noise” (Garrett et al., 2010). This stands in contrast to theories postulating that the brain is an intrinsically variable system, and that such variability may provide meaningful insights into its functional architecture (Stein et al., 2005; Faisal et al., 2008; Deco et al., 2011). Stemming from these emerging conceptualizations, novel approaches to analyzing rsfMRI data have been developed that focus on the moment-to-moment variability in the BOLD signal (Garrett et al., 2013b).

In recent years, an increasing number of studies have focused on BOLD signal variability in normative aging. For instance, a study by Garrett et al. (2010) examined the BOLD signal standard deviation (SD_BOLD_) in a sample of healthy adults ranging in age from 20 to 85 and found that patterns of resting-state SD_BOLD_ were generally more variable in younger adults. They suggested that this finding may reflect reductions in synaptic complexity and integrity in older age (Garrett et al., 2010). In support of this framework, subsequent task-based fMRI studies by the same group found that greater BOLD signal variability was associated with younger age and superior cognitive task performance (Garrett et al., 2011, 2013a, 2014; Grady and Garrett, 2014). Other studies have found greater resting-state SD_BOLD_ to be associated with increased microstructural integrity of white matter (WM) pathways in healthy older adults (Burzynska et al., 2015a,b). This relationship has not been a consistent finding, however, and some studies have regional *increases* in fMRI BOLD variance in older versus younger healthy adults (Garrett et al., 2010, 2011; Nomi et al., 2017), as well in individuals with stroke (Kielar et al., 2016), multiple sclerosis (Petracca et al., 2017) and other neurological disorders (Zöller et al., 2017). Thus, while the source of increased regional BOLD fluctuations remains unclear, it is conceivable that this may reflect sub-optimal functioning and/or compensatory mechanisms (Garrett et al., 2010; Nomi et al., 2017; Petracca et al., 2017). In general, the emerging consensus appears to be that variability in the rsfMRI BOLD signal may be a viable index of age-related cognitive decline that could provide new insights into age-related pathologies. Some rsfMRI studies have begun to examine other aspects of the temporal dynamics of spontaneous BOLD fluctuations in mild cognitive impairment (Han et al., 2011; Xi et al., 2012; Zhao et al., 2015) and AD (Liu et al., 2013, 2014), but the utility of variance measures such as SD_BOLD_ as a biomarker of AD requires further study.

Recently, Makedonov et al. (2013) used a slightly different measure of variability and found that BOLD fluctuations in WM regions are higher in (1) healthy older adults relative to younger adults, and (2) in the normal appearing WM structures of older adults with cerebral small vessel disease relative to healthy older adults (CSVD; Makedonov et al., 2013). The authors argued that increased arterial stiffness caused by cerebrovascular disease may result in greater pulsatility in vascular networks and small vessels, and result in the *increased* temporal variance of the BOLD signal observed in their study. Support for this suggestion was provided by a subsequent study that found greater variation of spontaneous BOLD fluctuations in both GM and WM in hypertensive elderly patients (Jahanian et al., 2014). Together, these findings suggest that resting-state BOLD variance may serve as a physiological signal related to an individual’s cerebrovascular status. Most recently, Makedonov et al. (2016) examined resting state in WM in individuals with AD and found significantly *increased* BOLD fluctuations in those with AD relative to those with mild cognitive impairment and to age-matched controls. Furthermore, they found that the increased BOLD fluctuations had a negative relationship with memory scores, thereby supporting a link between increased WM BOLD fluctuations and lower cognitive function.

The possibility that differences in BOLD fluctuations may reflect underlying differences in cerebrovascular health not captured by existing AD biomarkers that are associated with cognitive functioning, clearly warrants further study. To this end, we aimed to (1) examine whole brain differences in SD_BOLD_ in a group of individuals with AD and healthy age-matched controls, (2) determine whether measures of BOLD variability were related to measures of cognitive ability, and (3) investigate the relations between BOLD variability and WM cerebrovascular dysfunction. We hypothesized that there would be (1) widespread differences in rsfMRI BOLD variance in patients with AD versus healthy controls, (2) a relation between BOLD variability and participant cognitive test performance, and (3) a positive relation between BOLD variability and MRI-based measures of WM lesion burden.

## Materials and Methods

### ADNI Database

All data for the present study were obtained from the Alzheimer’s Disease Neuroimaging Initiative 2 (ADNI-2) database^1^. The ADNI, led by principal investigator Michael W. Weiner, began in 2003 as a partnership between the National Institute on Aging, the National Institute on Biomedical Imaging and Engineering, the Food and Drug Administration, as well as other private and public non-profit organizations. Since its launch, the primary goal of ADNI has been to develop more sensitive methods that may be able to detect AD at its earliest time point, in order to maximize the efficacy of future disease modifying or delaying interventions. Now in its fourth phase, the ADNI is focused on tracking the longitudinal progression of neuroimaging, laboratory, and neuropsychological AD biomarkers in participants from acquisition sites across Canada and the United States. For further information, please see^2^.

### Participants

All participants were selected from the ADNI-2 database, as the ADNI-1 phase did not collect rsfMRI data. In order to ensure the best variable control possible, inclusion to the AD group required (1) a clinician-confirmed diagnosis of mild AD at the screening visit, (2) complete rsfMRI data available for the first time point of the study. Given that ADNI participants are classified into groups (AD or “normal”) based on their clinical presentation at the screening visit, participants were selected from the first available time point (∼14 days post-screening) to ensure the continued accuracy of this diagnosis. The ADNI-2 database ultimately contained a total of 34 AD patients with rsfMRI data that met these criteria. Due to the novel methods used in the current study, extra caution was taken to ensure that the results of the analysis were not confounded by error-related noise: AD participants from this total pool were then manually selected for the current study based on a rigorous screening of raw data quality, as well as the accuracy of registration of the patient’s functional and structural images to standard stereotactic space, resulting in a final total of 19 participants in the AD group. An equivalent number of healthy control participants were then selected to form a comparable group. In the end, data were obtained from the first available time point from 19 individuals with AD (mean age = 72.7 years, *SD* = 6.5; 12 females) and 19 healthy age-matched controls (mean age = 74.7 years, *SD* = 6.9; 11 females). No significant differences were found between groups in participant age, sex, or education level. Participant demographic information can be found in **Table 1**.

**Table 1:** Participant demographics.

	AD	CN	AD vs. CN
Age	72.7 ± 6.5	74.7 ± 6.9	*p* = 0.365
Females	12	11	*p* = 0.740
Males	7	8	
Education (years)	16.2 ± 2.6	16.3 ± 2.3	*p* = 0.896

Diagnostic classification of AD participants was made by ADNI investigators according to diagnostic criteria for Probable AD established by the National Institute of Neurological and Communicative Disorders and Stroke and the Alzheimer’s Disease Related Disorders Association (NINCDS-ADRA; McKhann et al., 1984). Participants in the AD cohort also exhibited abnormal memory function on the Logical Memory II subscale of the revised Wechsler Memory Scale (WMS II, ≤8 for 16 years education and above), a Mini Mental State Exam (MMSE) between 20 and 26 (inclusive), and a Clinical Dementia Rating of 0.5 (very mild) or 1 (mild).

All control participants were free of memory complaints and deemed cognitively normal based on clinical assessments by the site physician showing an absence of significant impairment in cognitive functioning and performance of daily activities. Participants in the control cohort also exhibited normal memory function on the Logical Memory II subscale of the revised WMS (WMS II, ≥9 for 16 years of education and above), a MMSE score between 24 and 30 (inclusive), and a Clinical Dementia Rating of 0. For more information on group classifications, including all additional eligibility criteria, please consult the ADNI-2 procedures manual (Alzheimer’s Disease Neuroimaging Initiative, 2008).

All ADNI participants or their authorized representatives provided written informed consent approved by the Institutional Review Board at each acquisition site. For the purpose of the current study, secondary use of the data was approved by the Human Research Ethics Board at the University of Victoria (Victoria, BC, Canada).

### Image Acquisition

MRI data were downloaded with permission from the ADNI. All images were acquired on 3.0 Tesla Philips MRI scanners across 10 North American acquisitions sites according to the standardized ADNI protocol (Jack et al., 2008). Whole-brain anatomical MRI scans were acquired sagittally, with a T1-weighted MPRAGE sequence, with the following parameters: 1.2 mm slice thickness, 256 × 256 × 170 acquisition matrix, echo time (TE) of 3 ms, in-plane voxel dimension of 1 mm^2^, repetition time (TR) of 7 ms, and flip angle of 9°. Functional MRI scans were obtained during resting state; participants were instructed to lay quietly in the scanner with their eyes open. Resting state fMRI scans were obtained with a T2^∗^-weighted echo-planar imaging sequence with the following parameters: 140 volumes, 64 × 64 × 48 acquisition matrix (voxel size = 3.3 mm^3^), TE of 30 ms, TR of 3000 ms, and flip angle of 80°.

The participant’s T2-weighted fluid-attenuated inversion recovery (FLAIR) images were obtained for the purpose of lesion volume computation. The T2-weighted FLAIR images were obtained with 5.0 mm axial slices, a 256 × 256 × 35 acquisition matrix, a TE of 90 ms, an inversion time of 2500 ms, an in-plane voxel dimension of 0.85938 mm^2^, a TR of 9000 ms, and a flip angle of 90°.

### Data Analysis

#### Image Preprocessing

All analysis steps were performed using tools within the FSL version 5.0 (Analysis Group, FMRIB, Oxford, United Kingdom^3^; Smith et al., 2004). Non-brain tissue in the raw T1 images was removed using the automated Brain Extraction Tool (Smith, 2002), followed by manual verification and optimization for each subject. BOLD data preprocessing was performed in FSL’s FEAT as follows: each functional image was motion corrected and registered to their high-resolution T1 structural image that was linearly registered to standard stereotaxic space using a 12° of freedom transformation. A non-linear registration of the structural image to standard stereotactic space was also applied to account for potential local deformations in brains of the patient group. Additionally, global signal regression (GSR) was performed to correct for confounding physiological noise and to improve the detection of localized variation in the BOLD signal (Desjardins et al., 2001; Macey et al., 2004; Fox et al., 2009).

#### Statistical Comparisons

Individuals with AD were compared to age-matched controls in the group-level analysis. All results were examined at a *p* < 0.05 significance level, unless otherwise stated.

#### Resting-State BOLD Variability (SD_BOLD_)

Though different conceptualizations of brain signal variability exist, an increasingly popular approach in rsfMRI involves examining the distributional width of the neuroimaging timeseries by computing the signal variance or standard deviation (SD_BOLD_) across voxels (for a review, see Garrett et al., 2013b). Following this framework, the current study derived a measure of BOLD variability by first obtaining the variance of the residual signal left over after preprocessing at each voxel across the whole brain. The square root of the variance within each voxel was subsequently computed in order to arrive at a SD_BOLD_ map for each participant; this map effectively describes the standard deviation of the BOLD timeseries at each voxel within both GM and WM regions. The derived SD_BOLD_ maps from each individual were then merged into a single 4D file and smoothed with a Gaussian kernel (6 mm). To examine differences in resting state BOLD variability in AD patients versus healthy age-matched controls, between-group contrast comparisons of SD_BOLD_ were performed using randomize (Winkler et al., 2014) with threshold-free cluster enhancement and correction for multiple comparisons.

#### Relations between BOLD Variability and Cognitive Scores (Executive Function and Memory)

Neuropsychological data were obtained according to the protocol outlined in the ADNI-2 procedures manual (Alzheimer’s Disease Neuroimaging Initiative, 2008). The ADNI-2 database contains neuropsychological test scores for individual tests, as well as composite scores for the examination of memory and executive function. Deficits in both memory and executive function are core clinical characteristics of AD that have been shown to occur in the early stages of the disease (Baudic et al., 2006; Sperling et al., 2010). The use of composite measures, as opposed to individual cognitive tests, has been suggested to increase measurement precision and further limit the challenges associated with multiple hypothesis testing (Crane et al., 2012; Gibbons et al., 2012). In order to determine whether differences in resting state BOLD variability were related to participant cognitive test scores, correlations were examined between the participant’s SD_BOLD_ maps, as described above, and composite clinical scores for (1) memory performance (ADNI-MEM) and (2) executive function (ADNI-EF). Both of these cognitive measures have been derived from the ADNI neuropsychological test battery using item response theory (IRT) methods and validated in subsequent studies. Specifically, the ADNI-MEM score was derived from a single-factor confirmatory factor analysis model performed by Crane et al. (2012) using data from the Rey Auditory Verbal Learning Test (RAVLT), AD Assessment Schedule - Cognition (ADAS-Cog), MMSE, and the WMS Logical Memory subscale. The ADNI-EF score was derived by Gibbons et al. (2012) using a bi-factor confirmatory factor analysis model with data from the Wechsler Adult Intelligence Scale – Revised (WAIS-R) Digit Symbol Substitution, Digit Span Backward, Trails A and B, Category Fluency, and Clock Drawing. Due to missing data, two participants from each of the AD and CN groups were omitted from this analysis. Statistical computations were performed using randomize with threshold-free cluster enhancement and correction for multiple comparisons. Information on group cognitive performance can be found in **Table 2**.

**Table 2:** Participant clinical scores (separated by group).

	AD	CN	AD vs. CN
ADNI-MEM	–0.9516 ± 0.5250	0.8270 ± 0.4996	*p* = 0.000
ADNI-EF	–0.8996 ± 0.6935	0.7224 ± 0.4510	*p* = 0.000

#### Relations between BOLD Variability and Cerebrovascular Status (WM Lesion Volumes)

To derive a measure of cerebrovascular status, we identified participant white matter hyperintensities (WMHs) on FLAIR images and determined total WM lesion burden for each participant. Following data screening, four AD participants were omitted from this analysis due to FLAIR image motion artifact. Lesions were segmented by the lesion prediction algorithm (LPA; Schmidt, 2017), using FLAIR images only, as implemented in the LST toolbox version 2.0.15^4^ for the Statistical Parametric Mapping (SPM) software. Briefly, the LPA is a binary classifier in the form of a logistic regression model that was initially trained on the data of 53 multiple sclerosis patients with severe lesion patterns (Schmidt et al., 2012). Parameters of this model are used to segment lesions in new images by computing an estimate for the lesion probability within each voxel. In the current analysis, lesion volumes (ml) derived from each participant’s probabilistic map were extracted to derive a total lesion volume for each subject. To account for variability in parenchymal volume across participants, total brain volumes for each participant were computed using FMRIB’s Automated Segmentation Tool (FAST; Zhang et al., 2001). Lesion volumes were subsequently converted to a value representing the fraction of total brain volume (TBV) occupied by WMHs in each participant. Correlations were then examined between these values and the participants’ resting-state SD_BOLD_ maps. As in previous steps, statistical computations were performed using randomize with threshold-free cluster enhancement and correction for multiple comparisons.

#### Relations between WMH Burden and Cognitive Performance

As a secondary *post hoc* analysis, the relation between cognitive performance (ADNI-MEM and ADNI-EF) and WMH burden was examined across groups to better understand the contributions of cerebrovascular burden to the overall sample. Partial correlation coefficients were examined with two-tailed significance (*p* < 0.05), controlling for both age and education.

## Results

### Differences in Resting State SD_BOLD_ in Patients with AD versus Healthy Controls

**FIGURE 1:**
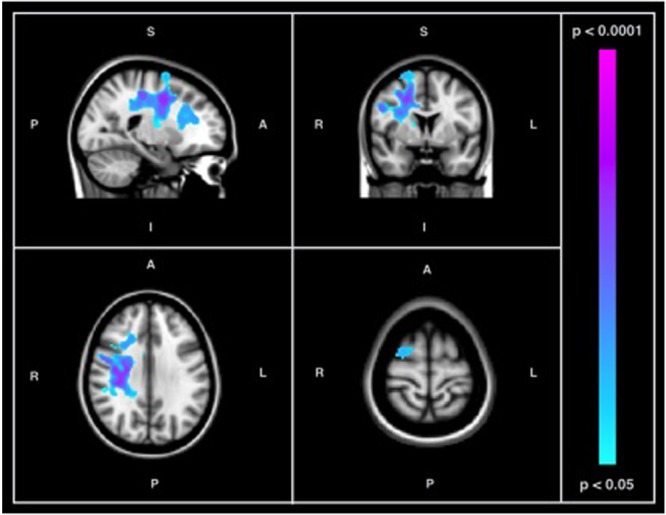
Results of between-group comparison of SD_BOLD_ in participants with AD versus healthy controls showing regions of increased signal variability in individuals with AD (*p* < 0.05, corrected for multiple comparisons) in both GM and WM. Images on overlaid on T1-weighted MNI152_T1_2mm standard template provided by the Functional MRI of the Brain’s Software Library.

Between-group comparisons were performed to examine differences in resting-state SD_BOLD_ in participants with AD relative to healthy age-matched controls. The results of this analysis revealed significant differences in BOLD variability (*p* < 0.05, corrected for multiple comparisons) in right-hemispheric GM and WM regions (**Table 3**). Specifically, AD > CN contrasts showed a greater resting state SD_BOLD_ in the AD group, predominantly in the right superior frontal gyrus and adjacent frontal regions, including the right precentral gyrus and right putamen. Significant WM regions were similarly right-lateralized and included portions of the right superior longitudinal fasciculus (frontal and temporal components) and right superior and inferior corona radiata (**Figure 1**). No GM or WM regions were found to have increased SD_BOLD_ in healthy controls relative to individuals with AD.

**Table 3:** Brain regions showing increased SD_BOLD_ in participants with AD relative to healthy controls (*p* < 0.05, corrected for multiple comparisons).

Brain region	Hemisphere	MNI coordinates		
		x	y	z
**Gray matter**				
Superior frontal gyrus	R	24	0	68
Precentral gyrus	R	56	4	32
Putamen	R	32	–8	0
**White matter**				
Superior longitudinal fasciculus	R	36	–22	28
Posterior corona radiata	R	24	–32	36
Superior corona radiata	R	24	–6	38
Anterior corona radiata	R	20	24	24
Forceps minor	R	20	38	16
External capsule	R	30	–8	12

*Coordinates are shown in Montreal Neurological Institute (MNI) standard stereotaxic space.*

### Relationship between Resting State SD_BOLD_ and Cognitive Scores

#### ADNI-MEM

**FIGURE 2:**
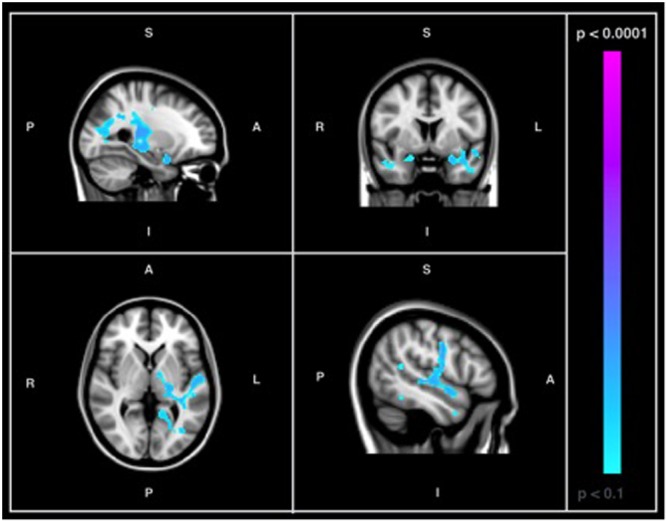
Images showing GM and WM regions where SD_BOLD_ is negatively associated with ADNI-MEM scores in healthy controls (*p* < 0.1, corrected for multiple comparisons). Images on overlaid on T1-weighted MNI152_T1_2mm standard template provided by the Functional MRI of the Brain’s Software Library.

When a conventional significance threshold (*p* < 0.05) was employed, no significant relationship was found between ADNI-MEM scores and SD_BOLD_ in either participants with AD or healthy controls. However, given the novelty and exploratory nature of this research, relationships were also explored using a more liberal threshold (*p* < 0.1, corrected for multiple comparisons). In doing so, an association was identified in the healthy control group, who demonstrated a negative relationship between SD_BOLD_ and ADNI-MEM scores in both GM and WM regions (**Table 4**). Specifically, lower ADNI-MEM scores were associated with greater SD_BOLD_ in the healthy control group in the left medial temporal lobe (MTL) and adjacent structures, including the right hippocampus and right amygdala, extending to the parahippocampal gyri bilaterally, as well as in the left superior longitudinal fasciculus and right inferior longitudinal fasciculus (**Figure 2**).

**Table 4:** Brain regions showing a negative association between SD_BOLD_ and ADNI-MEM scores in the healthy control group (*p* < 0.1, corrected for multiple comparisons).

Brain region	Hemisphere	MNI Coordinates		
		*x*	*y*	*z*
**Gray matter**				
Amygdala	R	20	0	–22
Hippocampus	R	22	–14	–24
Parahippocampal gyrus	R	22	4	–20
Parahippocampal gyrus	L	–16	–38	–14
Cerebellum	R	10	–50	–30
Cerebellum	L	–8	–50	–30
Temporal pole	L	–40	6	–24
Superior temporal gyrus	L	–64	–42	10
Planum temporale	L	–58	–12	4
Frontal orbital cortex	L	–28	8	–22
Heschl’s gyrus	L	–52	–14	6
Insular cortex	L	–40	–2	–8
Precuneus cortex	L	–14	–52	4
Thalamus	L	–12	–6	6
Lingual gyrus	L	–14	–52	2
**White Matter**				
Inferior longitudinal fasciculus	R	44	4	–28
Fornix/stria terminalis	L	–28	–20	–10
Superior longitudinal fasciculus	L	–42	–12	30
Superior corona radiata	L	–20	–16	34
Internal capsule (posterior limb)	L	–24	–22	8


*Coordinates are shown in Montreal Neurological Institute (MNI) standard stereotaxic space.*

#### ADNI-EF

No significant relationship was found between ADNI-EF scores and SD_BOLD_ in AD patients or healthy controls using either conventional or more liberal thresholds.

### Relations between Resting State SD_BOLD_ and Participant WMH Volume

**FIGURE 3:**
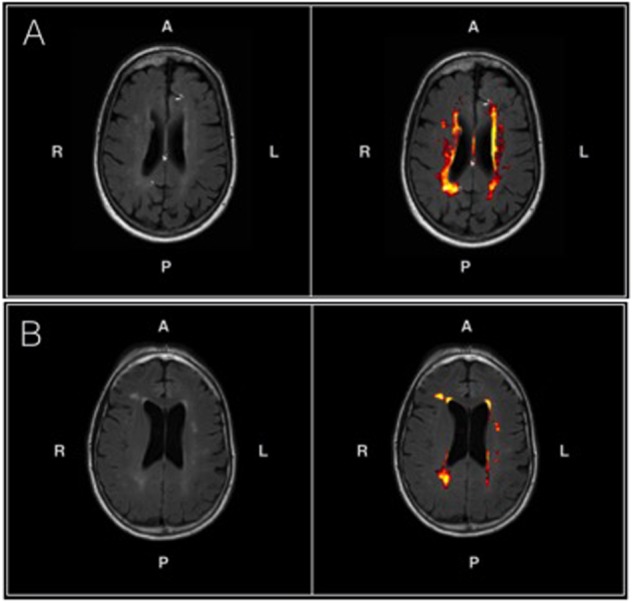
**(A)** An axial T2-FLAIR image of a prototypical patient from the AD group (left) and associated probabilistic lesion volume map (right) generated by the LST-LPA. **(B)** An axial T2-FLAIR image of a prototypical patient from the healthy control group (left) and associated probabilistic lesion volume map (right) generated by the LST-LPA. Brighter (yellow) hues indicate a higher lesion probability in that area, with lower lesion probabilities indicated by darker (red) hues. Prototypical AD and healthy control participants were selected based on the proximity of their WM lesion burdens to their respective group means.

**FIGURE 4:**
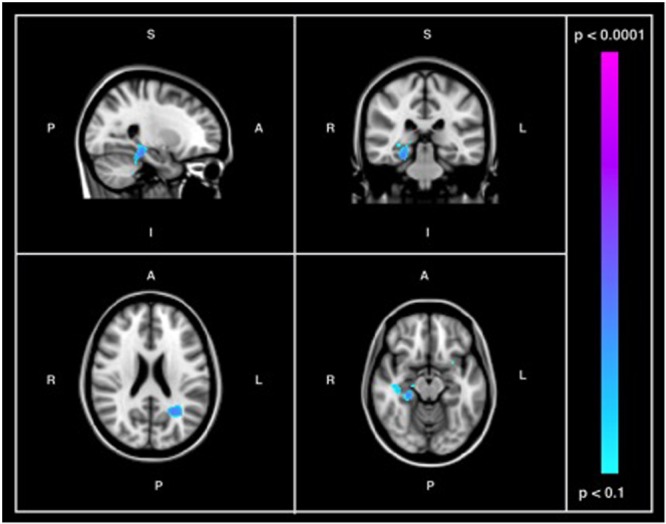
Images showing GM and WM regions where SD_BOLD_ is positively associated with WMH burden in the healthy control group (*p* < 0.1, corrected for multiple comparisons). Images on overlaid on T1-weighted MNI152_T1_2mm standard template provided by the Functional MRI of the Brain’s Software Library.

**Figure 3** shows the probabilistic lesion volume maps for a prototypical AD and healthy control participant. To account for total brain volume, lesion volumes were subsequently converted to a value representing the fraction of TBV occupied by WMH in each participant. On average, AD patients had higher total WM lesion burden (fractional lesion load = 0.011 ± 0.007) than age-matched controls (fractional lesion load = 0.007 ± 0.007). Using a conventional significance threshold (*p* < 0.05), no significant relation was found between total WMH burden and SD_BOLD_ in either group. However, using a more liberal threshold (*p* < 0.1, corrected for multiple comparisons) a relationship was identified in the healthy control group, which showed a positive association between WHM lesion burden and SD_BOLD_ in highly localized GM and WM regions (**Table 5**). Specifically, higher WMH lesion burden was associated with greater SD_BOLD_ in temporal, frontoparietal, and orbitofrontal regions, and most prominently in the right parahippocampal gyrus (**Figure 4**).

**Table 5:** Brain regions showing a positive association between SD_BOLD_ and white matter hyperintensity burden in the healthy control group (*p* < 0.1, corrected for multiple comparisons)

Brain region	Hemisphere	MNI coordinates		
		x	y	z
**Gray matter**				
Parahippocampal gyrus	R	26	–32	–14
Central opercular cortex	R	46	–14	12
Heschl’s gyrus	R	44	–14	8
Orbitofrontal cortex	L	–32	20	–26
**White matter**				
Posterior corona radiata	L	–28	–54	24

*Coordinates are shown in Montreal Neurological Institute (MNI) standard stereotaxic space.*

### Relations between WMH Burden and Cognitive Performance Across Groups

A significant negative correlation (*r* = -0.462, *p* < 0.01) was found between ADNI-MEM and WHM burden across participants, after correcting for age and education. Similarly, a significant negative relationship (*r* = -0.482, *p* < 0.05) was also found between ADNI-EF and WHM burden across participants, after correcting for age and education (**Table 6**).

**Table 6:** Partial correlation coefficients (*r*) for the relation between cognitive performance (ADNI-MEM and ADNI-EF) and WMH burden across groups, corrected for age and education.

	WMH Burden Corr. (r)	Significance
ADNI-MEM	–0.462	*p* = 0.008
ADNI-EF	–0.428	*p* = 0.015

## Discussion

The first goal of the current study was to examine and characterize differences in resting-state BOLD variability in AD relative to normative aging. In light of previous studies reporting a robust association between regional BOLD variability and age-related cognitive decline (Garrett et al., 2010, 2011, 2013a), we anticipated widespread differences in resting-state BOLD variability in patients with AD relative to healthy older adults. We observed significant increases in SD_BOLD_ in patients with AD in a number of GM and WM frontal regions, including portions of the superior frontal and precentral gyri, the superior longitudinal fasciculus, and widespread regions of the corona radiata (**Table 3**). Notably, both GM and WM regions with increased BOLD variability in AD were right lateralized (**Figure 1**). No brain regions were found to have increased SD_BOLD_ in healthy aging relative to AD.

The finding of increased BOLD variability in AD conflicts with earlier studies of SD_BOLD_ in normative aging, wherein greater BOLD variability has been posited to serve as a neural marker of optimal brain function (Grady and Garrett, 2014). Given the neurodegeneration inherent in AD, it is plausible to expect BOLD variability, when conceptualized as a neuronal signal of functional integrity, to be reduced in this population. However, studies have also revealed isolated regions of *increased* BOLD variability in older age (Garrett et al., 2010, 2011; Nomi et al., 2017), in stroke patients (Kielar et al., 2016) and in individuals with neurological disease (Petracca et al., 2017; Zöller et al., 2017), thereby alluding to a greater complexity the association between BOLD variability and functional integrity on a region-by-region basis. Notably, Makedonov et al. (2016), found that resting state BOLD fluctuations in WM were significantly increased in patients with AD relative to both participants with MCI and healthy controls. As a whole, it would appear that many of the studies in which increased BOLD fluctuations have been identified have examined patient populations. Given that brain signal variability has been found to exhibit robust age-related developmental trajectories (e.g., Grady and Garrett, 2014), it is therefore possible that aberrant findings in patient groups may reflect a disruption of normative brain processes.

However, a potential barrier to reconciling the current results with those found in previous studies, including the seminal studies by Garrett et al. (2010, 2011), relates to the lack of methodological standardization in this area of research. Indeed, a review of the recent literature shows that a number of different variations of ‘BOLD variability’ measures have been employed in previous studies, with different methodologies used by different groups to derive what are ostensibly the same variance measures, including SD_BOLD_. Though one of the aims of the current study was to increase transparency of the methodology used, this nonetheless remains a potential barrier to cross-study comparisons.

Another potential explanation for the current findings relates to the well-documented phenomenon of neural compensation in aging. In accordance with the present findings, many of the regions where increased BOLD variability has been identified in older or clinical populations have included portions of the frontal cortex (Petracca et al., 2017; Zöller et al., 2017), and particularly the superior frontal gyri (Garrett et al., 2010; Kielar et al., 2016). Interestingly, this localization to frontal regions is also reflected in mean-BOLD fMRI studies of aging, wherein prefrontal over-activation in older relative to younger adults has been postulated to reflect the additional recruitment of executive resources in support of memory maintenance (Reuter-Lorenz and Park, 2010). It is also notable that the right-hemispheric lateralization observed in the current study is also congruent with the existing BOLD fMRI compensatory literature: as summarized by Gauthier et al. (2013), one of the most common findings in aging is a decreased lateralization of the BOLD response, characterized primarily by a *decrease* in left frontal BOLD responses but an *increase* in the right frontal BOLD response (for a review see Reuter-Lorenz and Park, 2010). Extending these findings to the current study, it is therefore possible that similar mechanisms would apply in the resting-state, with the AD patients posing as an “accelerated aging” group relative to healthy aging. Keeping in mind the comparative-contrast methods in fMRI, this *decreased* frontal lateralization of in the AD group could plausibly have translated into an *increased* right frontal BOLD response when examining contrasts at the group level (i.e., AD – CN).

Given the present finding of increased signal variability in patients with AD relative to healthy controls, an important question remains: what might be the underlying psychophysiological correlates driving the increased signal variability in AD?

### SD_BOLD_ and Its Association with Cognitive Function

Central to the aforementioned compensation hypothesis, and reflected in previous findings of BOLD variability (e.g., Grady and Garrett, 2014), is the notion that SD_BOLD_ may serve as a neuronal index of cognitive function. Therefore, the second objective of the current study was to determine whether measures of BOLD variability might be associated with measures of memory and executive function (ADNI-MEM and ADNI-EF). Contrary to our hypothesis, we did not find a significant relationship between SD_BOLD_ and clinical test performance in patients with AD or healthy age-matched controls. Due to the preliminary nature of the present investigation, results were also examined with a more liberal threshold (*p* < 0.1). In doing so, an association was identified in the healthy control group, revealing a negative relationship between SD_BOLD_ and composite memory scores. Specifically, lower memory scores were associated with greater SD_BOLD_ in the healthy control group in the medial temporal lobe and adjacent structures (**Table 4**). No association was found between composite memory scores and SD_BOLD_ in the AD group. Moreover, no association was identified between composite scores of executive function and SD_BOLD_ in AD patients or healthy controls.

Though tentative interpretation is required, the trend toward a negative relationship identified in the healthy control group appears at odds with previous rsfMRI BOLD variability studies on healthy aging populations that have found that higher fluid intelligence and memory scores were linked to greater SD_BOLD_ in diffuse cortical regions (Burzynska et al., 2015b). However, these findings are more consistent with those by Makedonov et al. (2016), who examined patients with AD from the ADNI database and found that greater WM BOLD fluctuations were associated with *lower* composite scores of memory function in patients with AD, with no relationship identified between BOLD fluctuations and scores of executive function.

A number of factors may have contributed to the inconsistent association between BOLD variability and cognition observed in the present study. Notably, when Makedonov et al. (2016) included diagnostic status, glucose metabolism, and both whole brain and global nuisance signal regressors in a final omnibus model, composite memory scores were no longer found to be significantly associated with WM BOLD fluctuations. The authors postulate that WM BOLD fluctuations may therefore provide novel information about diagnostic status that is not captured by existing biomarkers, including cognition (Makedonov et al., 2016). Thus, it is possible that the trend-level association between SD_BOLD_ and memory observed in the control group merely reflects a corollary association between BOLD variability and another factor associated with memory composite scores in normative aging, that is otherwise disrupted by the diffuse pathophysiological changes in AD.

Another possibility for the present findings relates to the previous discussion on neural compensation in aging. If SD_BOLD_ indeed reflects compensatory activity, then it is reasonable to assume that there are likely limits to how effective that compensation can be. Indeed, it is possible that in AD such compensatory mechanisms are no longer effective, thereby rendering void any observable relationship between SD_BOLD_ and memory or executive abilities in the AD group. In contrast, the associations observed in normal aging may reflect active residual compensation effects.

However, it is also notable that many of the GM regions in which we found BOLD variability to be negatively associated (at trend-level) with composite memory scores in healthy controls included critical structures of the MTL known to subserve memory function, including the amygdala, hippocampus and parahippocampal gyri (Scoville and Milner, 1957; Cohen and Eichenbaum, 1993; Squire et al., 2004). In early AD, memory disturbances are often the primary and most salient clinical concern (Sperling et al., 2010). Moreover, in these early stages of the disease process, neuron loss figures most prominently in these MTL structures (Bottino et al., 2002). In addition to structural changes, up-regulatory functional changes are also known to occur in MTL memory circuits in patients with AD and in persons with mild cognitive impairment (Dickerson and Sperling, 2008; Faraco et al., 2013). Thus, the present findings suggest that resting state SD_BOLD_ may provide a novel method of characterizing changes in functional integrity in these regions.

In order to further elucidate the association between SD_BOLD_ in behaviorally relevant regions of the MTLs and its association with pathological aging, there is a need for additional longitudinal studies examining SD_BOLD_ and its association with specific measures of memory and cognition in prodromal aging groups with conversion to AD.

### SD_BOLD_ and Its Association with Cerebrovascular Health

Although cerebrovascular factors have previously been hypothesized to underlie BOLD fluctuation patterns in AD (Makedonov et al., 2016), this relationship had not been examined until the present study. Specifically, the third objective of the current study was to examine the association between GM and WM BOLD variability and neuroimaging markers of WM cerebrovascular burden. In light of the assertions by Makedonov et al. (2013, 2016), we hypothesized that there would be a positive association between resting-state BOLD variability and MRI-based measures of WM lesion load. When conventional thresholds were used, we did not find a significant association between total WMH burden and SD_BOLD_ in patients with AD or healthy controls. However, as in the previous analysis, we also chose to examine more liberal thresholds in order to further explore any potential directionality in the data. In doing so, an association was again identified in the healthy control group, showing a positive association between WMH lesion burden and SD_BOLD_. Specifically, participants with a higher WM lesion burden had greater SD_BOLD_ in a set of highly localized brain regions, among which most prominently included the right parahippocampal gyrus and right temporal cortex (**Figure 4**).

Though tentative, the present results are in line with the initial findings by Makedonov et al. (2013), who discovered that WM rsfMRI BOLD fluctuations were increased in the WM of patients with cerebral small vessel disease and positively correlated with WMH volume. This increased BOLD temporal variance has been suggested to reflect greater pulsatility in vascular networks and small vessels as a result of reduced cerebrovascular compliance (Makedonov et al., 2013, 2016). Other studies have also lent support to this vascular interpretation in both clinically hypertensive patients and healthy aging populations (Kannurpatti and Biswal, 2008; Kannurpatti et al., 2011; Jahanian et al., 2014).

Though the current results do not appear to support an association between resting-state BOLD variability and WMH lesion burden in AD, further considerations are warranted. Specifically, due to strict exclusion criteria necessitating a group of patients with AD without significant vascular comorbidity, the WHM lesion burden in ADNI appears relatively low (Ramirez et al., 2016), which may have contributed to underpowered effects. However, despite low WMH burden levels, there is evidence to suggest that they are non-trivial. For instance, Carmichael et al. (2010) found that WMH volumes at baseline predicted 1-year global cognitive decline in a sample of over 800 participants from the ADNI database. Moreover, consistent with this existing literature, results from our data suggest that greater vascular insult is uniformly associated with lower memory and executive functioning across the sample, even after correcting for age and education. This again echoes the need to re-examine the neurophysiological correlates of SD_BOLD_ from a longitudinal perspective.

Moreover, although the current study detected a trending relationship between vascular burden and BOLD signal variability in healthy controls, but not in patients with AD, the association between vascular risk factors and AD is one that is well established (O’Brien and Markus, 2014; Cai et al., 2015). Indeed, vascular risk factors, including hypertension and cholesterolemia, have been found to be significant risk factors for AD-specific neuropathology, including neuritic plaques and neurofibrillary tangles (Petrovitch et al., 2000; O’Brien and Markus, 2014). Interestingly, and in accordance with the results of the present study, a meta-analysis by Debette and Markus (2010) also found that WMHs, which are known markers of cerebrovascular outcomes (Debette and Markus, 2010; Chutinet and Rost, 2014), tended to be associated with an increased risk of dementia and AD in healthy populations, but *not* in patients who already exhibit cognitive impairment. In light of this evidence, examination of SD_BOLD_ as a non-invasive biomarker for underlying cerebrovascular risk factors in aging is one that directly contributes to the ultimate goal of improving the early identification of AD. For these reasons, further exploration of the link between rsfMRI SD_BOLD_ and vascular factors are needed, particularly in prodromal aging groups and with multimodal measures that may more comprehensively capture underlying cerebrovascular risk.

### Study Limitations

As discussed previously, one primary limitation of the current study relates to the diverse conceptualizations of fMRI BOLD variability, which have varied considerably across studies, making it challenging to systematically compare findings. Specifically, several different variations of ‘BOLD variability’ measures have been described (e.g., amplitude, variance, standard deviation, mean squared successive difference; for a review, see Garrett et al., 2013b), with considerable range in the methodology used to derive them. As argued by Garrett et al. (2013b), a significant barrier to adopting this novel imaging method on a larger scale lies in the lack of signal variability estimation tools in major neuroimaging software packages. Though some efforts have been made by Garrett et al. (2013b) to systematize the analysis of signal variability, implementation of BOLD variability as a novel imaging biomarker for AD will require increased efforts toward methodological standardization.

Another potential limitation of the current study is that only WMH burden was examined as a proxy for cerebrovascular status across groups. While WMHs are believed to be an indicator of cerebral small vessel disease (Mok and Kim, 2015) and cerebral arterial stiffness has found to be correlated with WHM lesion volume (Kidwell et al., 2001), they remain non-specific proxy measures. Future work should include more direct measures of cerebrovascular reactivity to re-examine the hypothesis that BOLD variability in neurodegenerative disease may reflect underlying cerebrovascular factors. Moreover, due to the strict exclusion criteria and, thus, the relatively low WMH lesion burden in the ADNI sample, future studies should examine more ecologically valid patient samples of AD with mixed vascular pathologies (Ramirez et al., 2016).

Finally, the sample size of the current study is small and limited to a single time point. To acquire a better understanding of BOLD variability and its association with AD pathology over time, future studies should examine larger samples at multiple time points of disease progression, which can be done in an ongoing manner as the ADNI dataset grows over time.

## Conclusion

Increasingly, AD has become an urgent public health concern, rendering critical the need to improve upon its early identification, so that disease-delaying treatments may be implemented as soon as they become available. In support of this goal, the current study examined a novel approach to the analysis of rsfMRI data. Though, traditionally, fMRI investigations have based findings on patterns of mean brain activity, moment-to-moment variability in the BOLD signal may provide new information on disorders of aging and their associated clinical correlates (Garrett et al., 2013b). The current study found increased SD_BOLD_ in patients with AD relative to healthy controls, with trends suggesting an association between SD_BOLD_ and both memory performance and WMH lesion burden in the control group. To further examine the clinical utility of this novel imaging parameter, future work should focus on longitudinal studies of SD_BOLD_ and its association with more comprehensive clinical and cerebrovascular data in both AD and prodromal aging.

## Author Contributions

VS was responsible for data analyses and drafting the manuscript. EM was responsible for designing the analyses. JF and LR were involved in interpretation of the results and critical revisions of the manuscript. JG supervised the project and was involved in data analyses, interpretation and critical revisions of the manuscript.

## Conflict of Interest Statement

The authors declare that the research was conducted in the absence of any commercial or financial relationships that could be construed as a potential conflict of interest. The reviewer RR and the handling Editor declared their shared affiliation.

## Footnotes

1 http://adni.loni.usc.edu

2 http://www.adni-info.org

3 http://fsl.fmrib.ox.ac.uk

4 http://www.statistical-modelling.de/lst.html

## Abbreviations

AD: Alzheimer’s disease
ADNI: Alzheimer’s Disease Neuroimaging Initiative
BOLD: blood oxygen level dependent
FSL: Functional MRI of the Brain Software Library
MRI: magnetic resonance imaging
MMSE: Mini Mental Status Exam
SD_BOLD_: standard deviation of the fMRI BOLD signal
WMH: white matter hyperintensity
WMS: Wechsler Memory Scale
